# Using Oncotype DX breast recurrence score® assay to define the role of neoadjuvant endocrine therapy in early-stage hormone receptor-positive breast cancer

**DOI:** 10.1007/s10549-023-06890-7

**Published:** 2023-03-10

**Authors:** Caitlin Taylor, Jane Meisel, Aimee J. Foreman, Christy Russell, Dipankar Bandyopadhyay, Xiaoyan Deng, Lisa Floyd, Amelia Zelnak, Harry Bear, Ruth O’Regan

**Affiliations:** 1 Ochsner Cancer Institute, New Orleans, LA USA; 2 Emory University, Atlanta, GA USA; 3 Exact Sciences Corporation, Madison, WI USA; 4 Massey Cancer Center, Virginia Commonwealth University, Richmond, VA USA; 5 Atlanta Cancer Care, Atlanta, GA USA; 6 University of Rochester, Rochester, NY USA

**Keywords:** Hormone receptor-positive breast cancer, Neoadjuvant therapy, Endocrine therapy, Oncotype, Recurrence Score

## Abstract

**Purpose:**

The role of neoadjuvant endocrine therapy in the treatment of patients with early-stage, hormone receptor-positive (HR +) breast cancer is not well defined. Tools to better determine which patients may benefit from neoadjuvant endocrine therapy versus chemotherapy or upfront surgery remain an unmet need.

**Methods:**

We assessed the rate of clinical and pathologic complete response (cCR, pCR) among a pooled cohort of patients with early-stage HR + breast cancer who had been randomized to neoadjuvant endocrine therapy or neoadjuvant chemotherapy in two earlier studies to understand better how outcomes varied by Oncotype DX Breast Recurrence Score® assay.

**Results:**

We observed that patients with intermediate RS results had no statistically significant differences in pathologic outcomes at the time of surgery based on whether they received neoadjuvant endocrine therapy or neoadjuvant chemotherapy, suggesting that a subgroup of women with a RS 0–25 may omit chemotherapy without compromising outcomes.

**Conclusion:**

These data suggest that Recurrence Score® (RS) results may serve as a useful tool in treatment decision-making in the neoadjuvant setting.

## Introduction

Adjuvant endocrine therapy remains the standard of care for patients with early-stage, HR + breast cancer who can safely omit chemotherapy based on RS results; however, the role of neoadjuvant endocrine therapy remains unclear [1, 2]. Historically, neoadjuvant endocrine therapy has been underutilized, especially in the US, with reported use as low as 3% among eligible patients [3]. Park et al. found that 46% of physicians reported using neoadjuvant endocrine therapy only rarely and 33% reported using sometimes [4]. Limited use of neoadjuvant endocrine therapy has stemmed from concerns regarding disease progression and unclear improvement in surgical outcomes, in addition to limited data on the optimal duration of treatment, endocrine therapy regimen, and the ideal patient population in terms of age and RS result [5–8].

The use of genomic assays has significantly impacted how physicians approach adjuvant treatment decisions for women with early-stage, HR + breast cancer; however, data for use in the neoadjuvant setting are limited. Tools to better determine which patients may benefit from neoadjuvant endocrine therapy versus neoadjuvant chemotherapy or upfront surgery followed by chemotherapy remain an unmet need. This secondary pooled analysis of randomized phase II prospective studies re-examines the use of neoadjuvant endocrine therapy among a cohort of premenopausal and postmenopausal patients with early-stage HR + , human epidermal growth factor receptor 2-negative (HER2-) breast cancer randomized to neoadjuvant endocrine therapy or neoadjuvant chemotherapy based on Oncotype DX® assay performed on initial core biopsy specimens. Performance of the 21-gene RS assay on biopsy specimens rather than tissue specimens following excision has demonstrated reliable results [9–11].

## Patients and methods

Data were pooled from two independent randomized phase II prospective studies of early-stage, HR + , HER2- patients with invasive breast cancer in a preoperative setting, performed at Emory University’s Winship Cancer Institute (Emory) and Massey Cancer Center at Virginia Commonwealth University (VCU) from 2010 to 2012; the latter included patients from 6 additional collaborating sites in the US and Canada [12, 13]. Data from the VCU analyses were previously published by Bear et al. and data from the Emory studies were presented as a poster at ASCO in 2013 [12, 13]. Eligibility criteria were also compared and consistent approaches to patient exclusions were verified.

The VCU multi-center study included women with HR + (defined as > 10% tumor staining by immunohistochemistry), HER2- invasive breast cancer measuring at least 2 cm and deemed by the surgeon not suitable for breast-conserving surgery unless size reduced by neoadjuvant therapy with ECOG performance status 0–1 [13]. Similarly, the Emory studies included women with confirmed early-stage (T1c-3, cN0-3, cM0), HR + , HER2- breast cancer with ECOG performance status 0–2 and no prior chemotherapy, hormonal therapy, or radiation [12]. Both studies assigned treatment based on the RS result: patients with RS result values of 0–10 received neoadjuvant endocrine therapy (Group A), those with intermediate values of 11–24 (Emory) or 11–25(VCU) were randomized to neoadjuvant endocrine therapy (Group B) or neoadjuvant chemotherapy (Group C), and patients with the highest RS result values of 25–100 (Emory) or 26–100 (VCU) received neoadjuvant chemotherapy (Group D).

Outcomes evaluated for this analysis include clinical partial and complete response (cPR, cCR) and pathologic complete response (pCR) in the breast and axillary nodes. Pathology reports from surgical specimens were reviewed for determination of pCR, and clinical partial and complete response were determined by review of radiology reports in the medical records using RECIST criteria. Of note, patients in the VCU cohort were deemed not suitable for breast conservation without tumor shrinkage as determined by a multidisciplinary treatment team.^13^

### Statistical analysis

Prior to combining the patients from each study into one analysis cohort, comparisons of the VCU and Emory patients were performed to assess whether the groups were similar enough for pooling. Due to small sample sizes, we were limited to conducting a simple descriptive exploration of study differences. Comparisons of continuous variables (age, RS result, chemotherapy cycles, months of endocrine therapy) were performed using the Wilcoxon Median 2-sample test, and categorical variables (race, ethnicity, menopausal status, nodal stage, HR status, grade, RS group, randomized neoadjuvant treatment group, cCR, cPR, and pCR) were evaluated using the Chi-square test or Fisher’s exact test for cell counts < 5. Comparisons could not be made by overall clinical stage, as this variable was not reported from VCU.

No clinically significant differences were identified that would limit pooling of the study data. Although event counts were too low to perform robust regression models predicting study outcomes, associations between RS result, type of neoadjuvant therapy (comparing all RS groups), and pCR in the breast, lymph nodes and breast plus lymph nodes were evaluated using Fisher’s exact test. Of note, one patient who did not receive Sentinel Lymph Node Biopsy (SLNB) or Axillary Lymph Node Dissection (ALND) was excluded from the denominator for pCR Nodes.

A sensitivity analysis was performed to assess the impact of the differing RS result cut-points in groups B and C between the studies on the outcomes. In this analysis, Emory patients with RS result = 25 were reassigned from Group D into Group C, since they were treated with neoadjuvant chemotherapy. Analyses were performed using SAS version 9.4 (SAS Institute, Cary, NC).

Both studies were designed as pilot studies to assess feasibility and subsequently, there were no pre-planned power and sample size estimates performed in the combined analysis. Post hoc power calculations are controversial and of limited utility.

## Results

**Fig. 1:**
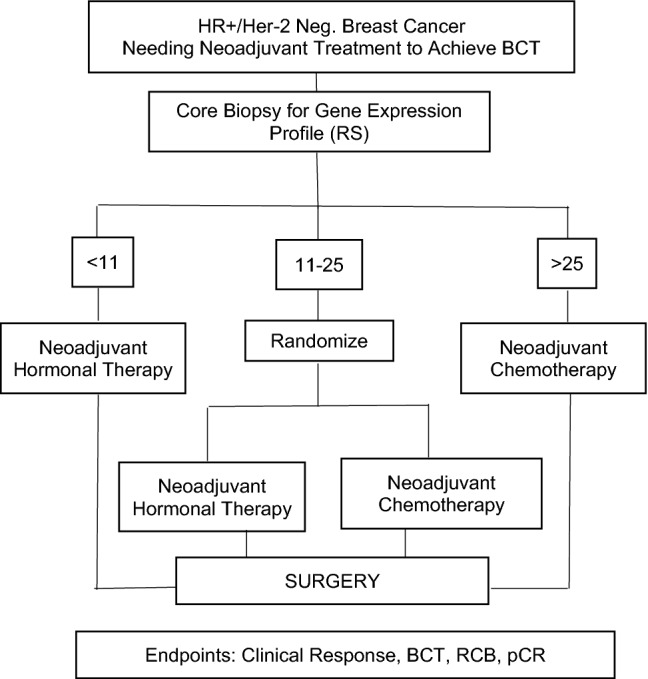
Protocol schema from VCU study [13]

**Fig. 2:**
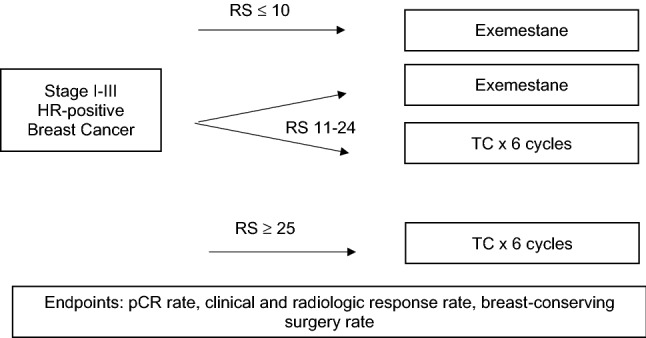
Protocol schema from Emory study [12]

**Fig. 3:**
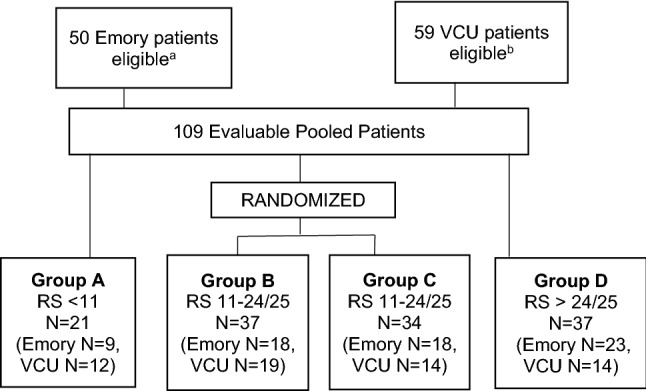
CONSORT diagram for combined **a** There were 4 patients initially enrolled in the Emory study who were ultimately excluded for the following reasons: two patients declined ongoing study participation due to their assigned treatment group; 1 patient elected to go off study due to low Oncotype DX result; 1 patient was treated off study due to a full neoadjuvant endocrine therapy arm. **b** 5 patients enrolled to the VCU study were ultimately excluded from study evaluation for the following reasons: 1 had delayed RS; 1 biopsy block was unable to be located; 1 hormone receptor discrepancy; 1 did not have a pregnancy test; 1 with insufficient tumor for RS

Our analysis included 109 eligible patients from both institutions (*n* = 50 from Emory, *n* = 59 from VCU). Protocol schema for the VCU and the Emory studies are shown below in Fig. 1 and Fig. 2, respectively. The CONSORT diagram for the combined data is shown in Fig. 3. The Emory cohort was younger, with a median age of 56 years vs 63 years in the VCU cohort (*p* = 0.015, Table 1). The Emory cohort was also more racially diverse, with 37.5% of patients being of African American ethnicity vs 18.6% in the VCU cohort (*p* = 0.029). Patients were predominantly postmenopausal (69.6% Emory vs 83.1% VCU, notably excluding 2 male patients from Emory, *p* = 0.103). Clinical nodal status among the Emory cohort was evenly divided, with 50% N0 and 50% N + , while the majority of VCU patients were N0 (76.3% N0 vs 22.0% N + , *p* = 0.004). Tumor grade was similar across the two cohorts, with the majority of patients in each group having Grade 2 tumors (60% Emory and 69% VCU, *p* = 0.340). Patients in the Emory study received longer courses of neoadjuvant endocrine therapy (median 10 months vs 5.5 months at VCU, *p* < 0.001), while patients in the chemotherapy treatment groups in both studies received a median of 6 cycles of treatment (*p* = 0.971). There were no significant differences in rates of cCR or pCR between study cohorts. Cohort characteristics are noted below in Table 1.

**Table 1:** Cohort characteristics

Variable	Emory (*N* = 50)	VCU (*N* = 59)	*P* value (a)
Age, median (range)	56 (35, 75)	64 (37, 80)	0.015
Race			
Caucasian	30 (62.5%)	48 (81.4%)	0.029
African American	18 (37.5%)	11 (18.6%)	
Female	48 (96.0%)	59 (100%)	0.208
Hispanic	1 (2.0%)	2 (3.4%)	1.000
Menopausal status (excludes 2 male patients in Emory cohort)			
Pre	14 (30.4%)	10 (16.9%)	0.103
Post	32 (69.6%)	49 (83.1%)	
N stage			
N0	25 (50.0%)	45 (76.3%)	0.004
N +	25 (50.0%)	13 (22.0%)	
NX	0 (0.0%)	1 (1.7%)	
Clinical stage^b^			
I	1 (2.0%)	N/A	N/A
II	43 (86.0%)	N/A	
III	6 (12.0%)	N/A	
Hormone receptor status, by IHC			
ER positive	50 (100.0%)	59 (100.0%)	N/A
PR positive	41 (82.0%)	52 (88.1%)	0.367
HER2 Negative, by IHC/FISH	50 (100.0%)	59 (100.0%)	N/A
Grade			
1	11 (22.0%)	13 (22.4%)	0.340
2	30 (60.0%)	40 (69.0%)	
3	9 (18.0%)	5 (8.6%)	
Oncotype Dx Recurrence Score, Median (range)	24 (2, 57)	18 (0, 65)	0.177
Oncotype Dx Recurrence Score Group			
< 11	9 (18.0%)	12 (20.3%)	0.042
11–24/25^c^	18 (36.0%)	33 (55.9%)	
24/25 +	23 (46.0%)	14 (23.7%)	
Randomized patient treatment			
Chemotherapy	11 (61.1%)	13 (39.4%)	0.138
Endocrine therapy	7 (38.9%)	20 (60.6%)	
Cycles of chemotherapy (among patients who received it), Median (range)	6 (1, 6)	6 (1, 12)	0.971
Months of endocrine therapy (among patients who received it), Median (range)	10.0 (6.0, 20.0)	5.5 (0.7, 8.5)	< .001
Outcomes			
cCR	7 (14.3%)	17 (28.8%)	0.071
cPR	16 (32.7%)	26 (44.1%)	0.226
pCR Breast	5 (10.2%)	4 (7.4%)	0.616
pCR Nodes	3 (6.1%)	3 (5.7%)	1.000
pCR breast + nodes	4 (8.2%)	4 (7.4%)	1.000

(a) The Wilcoxon Median 2-sample test was used for *P* value calculation for age, recurrence score, months of endocrine therapy, and cycles of chemotherapy. Fisher’s exact test was used for categorical variables with cell counts < 5 (Hispanic, N stage, HER2 negative) and the Chi-square test was used for other categorical variables (Race, Menopausal status, PR status, Grade, Oncotype Dx Recurrence Score Group, Randomized patient treatment)

(b) No overall clinical stage variable reported for VCU

(c) Intermediate RS values of 11–24 (Emory) or 11–25(VCU)

### RS groups

Patients were pooled and grouped based on RS result: RS < 11 (18.0% Emory and 20.3% VCU), RS 11–24 (Emory) or 25 (VCU) (36.0% Emory and 55.9% VCU), and RS 25 (Emory) or 26 (VCU) or higher (46.0% Emory and 23.7% VCU). Patients in Group A were older (median 64 years vs 59 years in Group D), with a higher percentage of low-grade tumors (47.6% grade 1 vs 5.4% grade 1 among RS > 24/25 in Group D). Patients with high RS result (Group D) had a higher percentage of nodal involvement (48.6%) than those with low RS result (23.8% in Group A). Nodal involvement among randomized patients with intermediate RS results differed somewhat, with 22.2% N + in Group B and 37.5% N + in Group C. Results are summarized in Table 2.

**Table 2:** Demographic and clinical characteristics by treatment group

Variable	Group A (*N* = 21)	Group B (*N* = 27)	Group C (*N* = 24)	Group D (*N* = 37)	*P* value (a)
Age, Median (range)	64 (38, 76)	59 (37, 80)	57 (40, 75)	59 (35, 72)	0.013
Race					
Caucasian	15 (71.4%)	23 (85.2%)	18 (75.0%)	22 (62.9%)	0.270
African American	6 (28.6%)	4 (14.8%)	6 (25.0%)	13 (37.1%)	
Hispanic	1 (4.8%)	0 (0.0%)	1 (4.2%)	1 (2.8%)	0.788
Menopausal status					
Pre	3 (14.3%)	6 (22.2%)	9 (37.5%)	6 (18.2%)	0.240
Post	18 (85.7%)	21 (77.8%)	15 (62.5%)	27 (81.8%)	
N stage					
N0	16 (76.2%)	20 (74.1%)	15 (62.5%)	19 (51.4%)	0.127
N +	5 (23.8%)	6 (22.2%)	9 (37.5%)	18 (48.6%)	
NX	0 (0.0%)	1 (3.7%)	0 (0.0%)	0 (0.0%)	
Hormone receptor status, by IHC					
ER positive	21 (100.0%)	27 (100.0%)	24 (100.0%)	37 (100.0%)	N/A
PR positive	19 (90.5%)	23 (85.2%)	21 (87.5%)	30 (81.1%)	0.785
HER2 Negative, by IHC/FISH	21 (100.0%)	27 (100.0%)	24 (100.0%)	37 (100.0%)	N/A
Grade					
1	10 (47.6%)	9 (34.6%)	3 (12.5%)	2 (5.4%)	0.0002
2	11 (52.4%)	14 (53.8%)	20 (83.3%)	25 (67.6%)	
3	0 (0.0%)	3 (11.5%)	1 (4.2%)	10 (27.0%)	
Oncotype Dx Recurrence Score, Median (range)	8 (0, 10)	19 (11, 25)	18 (11, 24)	33 (25, 65)	< .001
Oncotype Dx Recurrence Score Group					
< 11	21 (100.0%)	0 (0.0%)	0 (0.0%)	0 (0.0%)	N/A
11–24/25	0 (0.0%)	27 (100.0%)	24 (100.0%)	0 (0.0%)	
24/25 +	0 (0.0%)	0 (0.0%)	0 (0.0%)	37 (100.0%)	

(a) Nonparametric testing was used for *P* value calculation for age, recurrence score, months of endocrine therapy, and cycles of chemotherapy. Fisher’s exact test was used for categorical variables with cell counts < 5

Group A = Recurrence Score < 11, Group B = Recurrence Score 11–24 (Emory study) or 11–25 (VCU study) receiving NHT, Group C = Recurrence score 11–24 (Emory study) or 11–25 (VCU study) receiving NCT, and Group D = Recurrence score > 24 (Emory study) or > 25 (VCU study)

Among patients with high RS result in group D, 2 Emory patients (8.7%) achieved cCR versus 4 patients (28.6%) in the VCU group (*p* = 0.1735). Rates of cCR among patients with intermediate and low RS varied between groups with 23.5% (Emory) vs 36.4% (VCU) achieving cCR with intermediate-risk scores (Groups B and C), and 11.1% (Emory) vs 8.3% (VCU) achieving cCR with low RS in Group A (results not shown).

### Rates of pCR

With regard to pCR, there were few events reported among patients with low or intermediate RS results in the pooled data. One patient (4.8%) in Group A was found to have pCR in the breast plus lymph nodes (4.8%), while no patients in either randomized group achieved pCR in the breast or breast plus lymph nodes. While there were different rates of pCR in the lymph nodes in the randomized groups (4.3% in Group B vs 13.6% in Group C), they did not reach statistical significance (*p* = 0.298). Patients with high RS results (Group D) were shown to have significantly higher rates of pCR in breast plus lymph nodes across groups (18.9%, *p* = 0.014). Notably, while patients on the Emory study received longer courses of neoadjuvant endocrine therapy (median 10 months at Emory vs 5.5 months at VCU), there were no significant differences in pCR across RS result subgroups noted between the two institutions. Table 3 summarizes rates of pCR according to treatment groups.

**Table 3:** pCR according to treatment groups (All Eligible Patients)

Variable	Group A (*N* = 21)	Group B (*N* = 23)	Group C (*N* = 22)	Group D (*N* = 37)	*P* value (a)
pCR Breast	1 (4.8%)	0 (0.0%)	0 (0.0%)	8 (21.6%)	0.0059
pCR Nodes	0 (0.0%)	1 (4.3%)	3 (13.6%)	2 (5.6%)	0.2977
pCR Breast + Nodes	1 (4.8%)	0 (0.0%)	0 (0.0%)	7 (18.9%)	0.0143

(a) Fisher’s exact test was used for categorical variables with cell counts < 5

Group A = Recurrence Score < 11, Group B = Recurrence Score 11–24 (Emory) or 11–25 (VCU) receiving NET, Group C = Recurrence Score 11–24 (Emory) or 11–25 (VCU) receiving NCT, and Group D = Recurrence Score > 24 (Emory) or > 25 (VCU)

Since the one Emory patient with RS result 25 did not experience pCR, sensitivity analyses re-categorizing them to group C only impacted the denominators. Significance for differences did not shift (results not shown).

## Discussion

In this study, we observed that patients with intermediate RS results had no statistically significant differences in pathologic outcomes at the time of surgery based on whether they received neoadjuvant endocrine therapy or neoadjuvant chemotherapy. While our study did not include secondary analyses of pCR based on lymph node involvement or menopausal status among RS groups, the results suggest that a subgroup of women with a RS 0–25 may omit chemotherapy without compromising rates of clinical and pathologic complete response. This aligns with data from the Trial Assigning Individualized Options for Treatments (TAILORx), establishing no benefit of chemotherapy among women over the age of 50 years with HR + node-negative (N0) breast cancer and RS 11–25, as well as RxPONDER, which demonstrated that postmenopausal women with HR + node-positive (1–3 positive nodes) breast cancer and RS ≤ 25 can safely avoid chemotherapy [1, 14].

Our study findings suggest that the RS result may be an impactful tool to help guide treatment decisions in the neoadjuvant setting and reinforces the clinical feasibility of the RS assay using core biopsy specimens. The use of NET, which has been more often used in Europe than in the US, is a reasonable approach to downstaging some HR + tumors to allow de-escalation of surgery, particularly to allow breast conservation that might otherwise not be possible. Currently validated for use in the adjuvant setting, gene expression profiles like the Oncotype DX assay and the 70-gene signature MammaPrint assay have significantly contributed to better understanding of the heterogeneity among early-stage HR + breast cancer and, in doing so, have decreased the number of women exposed to the potential toxicities of chemotherapy [15]. As the quest for precision care continues, the far-reaching potential for application of gene expression profiles to varying subgroups of women in the neoadjuvant setting remains an active area of interest.

Ongoing trials evaluating the use of neoadjuvant endocrine therapy in combination with cyclin-dependent kinase (CDK) 4/6 inhibitors have shown promising results [8, 16, 17]. While the combination of CDK4/6 inhibitors and endocrine therapy prolongs progression-free survival and overall survival among patients with metastatic HR + breast cancer and more recently has demonstrated clinical benefit in patients with HR + N + high risk early breast cancer, the potential benefit of these combinations in the neoadjuvant setting remains unclear [18–21]. Phase II data from the NeoPalAna trial conclude that the combination of neoadjuvant palbociclib and anastrozole induces a higher rate of complete cell-cycle arrest (defined as Ki67 ≤ 2.7%) than anastrozole alone in patients with clinical stage II/III HR + breast cancer [16]. The neoMONARCH trial evaluating the biological and clinical activity of combined neoadjuvant abemaciclib and anastrozole in postmenopausal women with stage I-IIIB HR + /Her2- breast cancer demonstrated increased rates of complete cell-cycle arrest and decrease in Ki67, with 46% of intention-to-treat patients achieving a radiologic response [17]. These data have supported ongoing studies further evaluating the use of CDK4/6 inhibitors and endocrine therapy combinations in the neoadjuvant setting. Our study successfully showed the utility of the Oncotype DX assay in the neoadjuvant setting and highlights the feasibility and potential clinical impact of its application in ongoing research of neoadjuvant CDK4/6 inhibitors and endocrine therapy.

Finally, it is important to note the difference in duration of neoadjuvant endocrine therapy between the two institutions. Despite patients in the Emory cohort receiving significantly longer courses of neoadjuvant endocrine therapy (median 10 months versus 5.5), there were no significant differences in pCR across RS result groups between the two institutions, and nearly three quarters of the VCU patients were able to receive breast-conserving treatment despite the pre-treatment assessment that they would require total mastectomies. Duration of neoadjuvant endocrine therapy has historically ranged from 12 to 24 weeks, although adequate duration to optimize outcomes has not been defined [8]. One study evaluating the impact of neoadjuvant endocrine therapy on objective response rate (ORR) found no significant differences between short (< 9 weeks), moderate (9–27 weeks), and long (> 27 weeks) durations of treatment (ORR was 56.7%, 52.1%, and 49%, respectively) [22]. In their phase II study evaluating neoadjuvant endocrine therapy versus neoadjuvant chemotherapy in postmenopausal women with HR + breast cancer, Semiglazov et al. noted a median time to clinical response of 57 days in patients receiving endocrine therapy [23]. Data from the Edinburgh Breast Unit demonstrate high response rates with reductions in tumor volume greater than 80% in some cases of postmenopausal women with estrogen receptor-rich tumors after just three months of NET, while Fontein et al. demonstrated improved clinical response and breast conservation rates following six months of neoadjuvant endocrine therapy when compared to three [24, 25]. These findings suggest that when used in the appropriate patient population, the duration of neoadjuvant endocrine therapy may be adjusted pending other treatment considerations and clinical response. Ultimately, recommendations for neoadjuvant endocrine therapy should be individualized, taking into consideration clinical response, potential side effects, surgical planning, and patient preference.

Limitations of this study include the narrow scope in assessing pCR, lack of long-term follow-up and limited number of premenopausal patients. We did not assess the differences in side effects (both immediate or long-term) between treatment groups, nor did we evaluate for differences in disease recurrence. While the difference in RS result groupings (11–24 for Emory, 11–25 for VCU) created an inconsistency between studies, the sensitivity analysis illustrated that it had no impact on the results. Due to the low number of patients as described above and events for each study, we were unable to provide more than a descriptive explanation of the study differences. We explored adjusted analyses accounting for random effects of study and fixed effects for variables including length of endocrine therapy to determine the impact of differences between studies on pooling the data, but were unable to achieve model convergence. Finally, secondary analyses of data from previously designed studies can be subject to bias, even though these two studies had similar objectives. Despite these limitations, this study importantly highlights the potential for the Oncotype DX assay use in the neoadjuvant setting for therapeutic decision-making and the impact this may have on further personalizing treatment plans for a large group of patients with breast cancer.

## Conclusion

Our results demonstrate that the use of the Oncotype DX Breast Recurrence Score assay in the neoadjuvant setting may help guide treatment decisions when considering the use of neoadjuvant endocrine therapy versus neoadjuvant chemotherapy. Patient age and planned duration of endocrine therapy as well as patient preferences should be considered when determining neoadjuvant treatment plans. Ongoing studies evaluating the use of neoadjuvant endocrine therapy with CDK4/6 inhibitors will offer further insight into optimal neoadjuvant treatment strategies in HR + breast cancer [16, 17, 26]. Subsequent phase III evaluation of the role of genomic assays in the neoadjuvant setting is feasible and may help determine whether neoadjuvant endocrine therapy + CDK 4/6 inhibitors could replace neoadjuvant chemotherapy for patients with higher RS values.

## Data Availability

Enquiries about data availability should be directed to the authors.
